# Assessment of dietary-lifestyle patterns and adherence to the USDA recommendations in Lebanese pregnant women amid the economic crisis: Findings from a national representative cross-sectional study

**DOI:** 10.1371/journal.pone.0301170

**Published:** 2024-04-11

**Authors:** Maha Hoteit, Rana Mahfouz, Yonna Sacre, Sara Obeid, Mariane Abou Nasr, Mohamad El Hajj, Lara Hanna-Wakim, Chadi Fakih

**Affiliations:** 1 Food Sciences Unit, National Council For Scientific Research (CNRS-L), Beirut, Lebanon; 2 PHENOL Research Group (Public Health Nutrition Program Lebanon), Faculty of Public Health, Lebanese University, Beirut, Lebanon; 3 Department of Nutrition and Food Sciences, Faculty of Arts and Sciences, Holy Spirit University of Kaslik (USEK), Jounieh, Lebanon; 4 Department of Agricultural and Food Engineering, School of Engineering, Holy Spirit University of Kaslik (USEK), Jounieh, Lebanon; 5 Al Hadi Laboratory and Medical Center, Beirut, Lebanon; 6 Department of Obstetrics and Gynecology, Faculty of Medicine, Lebanese University, Beirut, Lebanon; University of Petra (UOP), JORDAN

## Abstract

As Lebanon’s economic crisis become uncontrollable, Lebanese pregnant women face malnutrition, with many having to skip meals and switch to resort to cheap and unhealthy alternatives altogether. The objectives of the study were to assess the dietary and lifestyle patterns of Lebanese pregnant women and to evaluate their diets compliance with the United States Department of Agriculture (USDA) pregnancy recommendations, before and during the Lebanese escalating economic crisis. A cross-sectional study was conducted between April 2021 and January 2022. A validated self-administrated questionnaire was administered during the first, second and third trimesters of pregnancy among 363 women in all Lebanese governorates. Most of the pregnant women were free of diseases. While the majority did not smoke, 14.1% smoked hookah / shisha during pregnancy. The adherence to the USDA recommendations in our sample did not significantly vary prior to and throughout the socioeconomic crisis, and it was generally low. Only the mean consumption of vegetables increased during the socioeconomic crisis (p<0.05). Regarding physical activity, while the proportion of active women slightly decreased during the socioeconomic crisis, around 55% were still active. In conclusion, higher attention should be given to the dietary habits and health of this critical population, through effective interventions that increase awareness and achieve measurable improvements.

## Introduction

Following a series of global shocks, most nations around the world, including those in the Middle-East/North Africa (MENA) area, were struggling. For almost four years, Lebanon has been experiencing the worst, multifaceted socio-economic catastrophe. The concurrent effects of the Corona Virus of 2019 (COVID-19) breakout and the enormous Port of Beirut explosion in August 2020 have further aggravated the ongoing economic and financial crisis that began in October 2019 [1]. Consequently, thousands of Lebanese households have been pushed into poverty and unemployment. According to United Nations (UN) agencies, two million people in Lebanon, including 1.29 million Lebanese citizens are currently facing some level of food insecurity [2] within adequate supply of, and access to, safe and nutritious food that fits their dietary needs and food choices for an active and healthy life [3]. Food prices in Lebanon has soared by 239 percent over the past four years, which has threaten the pregnant women’s health, with the majority either having to rely on the cheapest alternatives or having to skip meals [4].

Thus, due to greater nutritional needs during pregnancy, women experience a higher risk of emergencies [5]. It is essential to prevent malnutrition by providing pregnant women with the necessary dietary support and guidelines [6]. To prevent possible nutritional deficiencies, many nutrition-related organizations and professionals encourage to adapt the dietary guidelines and ensure a high adherence to the five-food group-focused USDA dietary recommendations [7].

In Lebanon, understanding eating habits and the degree of adherence to dietary recommendations will help increase awareness and develop future policies and practices focused at improving dietary behaviors during pregnancy. Therefore, the objective of this study is to investigate the dietary patterns, and adherence to pregnancy-related USDA guidelines among Lebanese pregnant women before and throughout the socio-economic crisis.

## Materials and methods

### Study population

This research is of a cross-sectional type performed during the socioeconomic crisis. Participants were 363 pregnant women recruited from all Lebanese governorates. The Lebanese University’s Ethics Committee in Scientific Research (CUER#30–2020) approved the study design. To qualify for participation in the study, women had to be aged >18 years, pregnant since pre-crisis period and having normal pregnancy. Participants consented to participate and replied to all questions. Besides, women who got pregnant prior to the socioeconomic crisis and those who had plausible risk factors during pregnancy were excluded from the study.

### Data collection

A self-administrated pretested and validated questionnaire was distributed via social media platforms [8, 9]. (Accessible at https://doi.org/10.17605/OSF.IO/NP973). Answers were anonymous and confidential. The questionnaire was used to describe dietary patterns, consumption, and compliance with the United States Department of Agriculture (USDA) pregnancy recommendations among Lebanese pregnant mothers prior to and throughout the socioeconomic crisis period. Several sections on the form provided questions on sociodemographic information, medical history, food preferences, physical activity, biometric information, tobacco use, stress, and depressive symptoms. Pregnant women’s evolution, daily serving sizes for the main dietary groups, mental well-being, tobacco use, and levels of exercise were all evaluated by the questionnaire.

### Measurements and scales

#### Body mass index

To determine the body mass index (BMI) prior to pregnancy, the WHO standards were followed [10].

#### Physical activity

Pregnant women who reported being engaged for a minimum of 30 minutes each day at any intensity (light, moderate, or vigorous) were classed as being active [11].

#### Dietary recommendations for pregnant women

According to the USDA’s guidelines for expectant women, recommended serving sizes for various foods were categorized [12]. According to the USDA’s daily guidelines, each food category was given a score of 0 or 1, with 0 suggesting a lower intake and 1 indicating a larger intake.

Healthy eating while pregnant consists of consuming the recommended servings for pregnancy:

Bread, rice, and cereals (**≤**6 servings: low intake; **≥**6 servings: high intake)

Fruits (**≤** 2 servings; low intake; **≥**2 servings: high intake)

Vegetables (**≤**2.5 servings: low intake; **≥**2.5 servings: high intake)

Proteins (**≤**5.5 servings: low intake; **≥**5.5 servings: high intake)

Dairy (**≤**3 servings: low intake; **≥**3 servings: high intake)

The score for adherence was calculated by adding together the compliance with each dietary category. Subsequently, poor adherence score (0–2) and increased adherence score categories were assigned to evaluate adherence.

### Statistical analysis

Continuous variables were expressed in mean+SD, and categorical ones in frequencies and percentage. Non-parametric Related-samples Wilcoxon Signed Rank Test and non-parametric Related-samples McNemar Test were performed for scale and categorical variables respectively, to conduct comparisons before versus during the socioeconomic crisis. Chi square test and independent samples T-Test were performed for categorical variables to conduct comparisons across governorates. Non-parametric Independent-samples Kruskal-Wallis Test and non-parametric Independent-samples Mann-Whitney U Test were performed instead of the ANOVA and independent-samples T-test respectively to analyze the effect of dietary habits on adherence to USDA dietary guidelines. P<0.05 was considered significant. Statistical analysis was done using IBM SPSS version 23.

## Results

### Characteristics of the study sample

Table 1 represents the socio-economic and health characteristics of the study participants (n = 363), overall and by governorates. Around 75% of the women in this study aged 25 years and above, with majority having higher degrees. Half the women in this study were unemployed and half of the sample complained about family income loss due to the financial meltdown. Around 70% of women were in their third trimester, 35% had overweight and almost the majority were non-smoker and free of chronic diseases. As for physical activity, pregnant women in this study reported a decrease of 75% in comparison with the period preceding the economic crisis (Table 1).

**Table 1:** Socio-economic and health characteristics of the study participants (n = 363), overall and by governorates.

	Total Sample	Beirut	Mount Lebanon	North Lebanon	South Lebanon	Nabatieh	Beqaa	Baalbek-Hermel	p-Value
	**N (%) 363**	145 (39.9)	63 (17.4)	16 (4.4)	67 (18.5)	44 (12.1)	9 (2.5)	19 (5.2)	
**Age (mean ± SD)** ^**a**^	28.34±4.85	27.94± 4.86	30.30± 4.46	31.69± 3.66	28.75± 4.46	26.16± 5.12	27.22± 5.61	26.32± 3.64	0.000
**Age** ^**b**^									0.005
Youth 18–24 years	91 (25.1)	38 (26.2)	11 (17.5)	0	13 (19.4)	17 (38.6)	5 (55.6)	7 (36.8)	
Young adults ≥25 years	272 (74.9)	107 (73.8)	52 (82.5)	16 (100.0)	54 (80.6)	27 (61.4)	4 (44.4)	12 (63.2)	
**Education level** ^**b**^									0.155
High School or less	39 (10.7)	13 (9.0)	3 (4.8)	3 (18.8)	10 (14.9)	8 (18.2)	0	2 (10.5)	
Diploma	82 (22.6)	29 (20.0)	9 (14.3)	6 (37.5)	19 (28.4)	12 (27.3)	3 (33.3)	4 (21.1)	
Bachelor’s degree	142 (39.1)	59 (40.7)	30 (47.6)	2 (12.5)	25 (37.3)	15 (34.1)	2 (22.2)	9 (47.4)	
Postgraduate studies	100 (27.5)	44 (30.3)	21 (33.3)	5 (31.3)	13 (19.4)	9 (20.5)	4 (44.4)	4 (21.1)	
**Employment status** ^**b**^									0.001
Employed	169 (46.6)	74 (51.0)	41 (65.1)	4 (25.0)	26 38.8)	11 (25.0)	5 (55.6)	8 (42.1)	
Unemployed	194 (53.4)	71 (49.0%)	22 (34.9)	12 (75.0)	41 61.2)	33 (75.0)	4 (44.4)	11 (57.9)	
**Family income during the crisis** ^**b**^									0.216
Decreased	181 (49.9)	75 (51.7)	41 (65.1)	8 (50.0)	30 (44.8)	17 (38.6)	2 (22.2)	8 (42.1)	
Increased	33 (9.1)	13 (9.0)	1 (1.60)	2 (12.5)	8 (11.9)	5 (11.4)	1 (11.1)	3 (15.8)	
No change	149 (41.0)	57 (39.3)	21 (33.3)	6 (37.5)	29 (43.3)	22 (50.0)	6 (66.7)	8 (42.1)	
**Pregnancy Trimester**^**b**^									0.484
Trimester 1	35 (9.64)	10 (6.89)	6 (9.5)	3 (18.7)	5 (7.46)	8 (18.8)	2 (22.2)	1 (5.26)	
Trimester 2	43 (11.85)	16 (11.0)	10 (15.87)	2 (12.5)	9 (13.4)	3 (6.81)	0	3 (15.78)	
Trimester 3	220 (60.6)	88 (60.68)	41 (65.0)	7 (43.75)	42 (62.68)	25 (56.8)	7 (77.8)	10 (52.6)	
choose not to respond	65 (17.9)	31 (21.37)	6 (9.5)	4 (25)	11 (16.4)	8 (18.18)	0	5 (52.6)	
**Pre-pregnancy BMI**^**a**^	24.27±4.63	24.40± 4.68	24.10± 4.11	25.04± 7.26	24.42± 4.41	24.36± 4.56	21.27± 1.67	24.36± 5.98	0.634
**Overweight/Obese**^**b**^									0.586
Normal body Weight	190 (52.3)	75 (51.7)	38 (60.3)	7 (43.7)	34 (50.7)	19 (43.18)	8 (88.9)	9 (47.3)	
Overweight/Obese	103 (28.3)	39 (26.8)	18 (28.5)	5 (31.2)	20 (29.8)	16 (36.3)	1 (11.1)	4 (21)	
choose not to respond	70 (19.2)	31 (21.3)	7 (11.1)	4 (25)	13 (19.4)	9 (20.4)	0	6 (31.5)	
**Gestational diabetes** ^**b**^									0.231
No	353 (97.2)	142 (97.9)	60 (95.2)	14 (87.5)	66 (98.5)	43 (97.7)	9 (100)	19 (100)	
Yes	10 (2.8)	3 (2.1)	3 (4.8)	2 (12.5)	1(1.5)	1 (2.5)	0	0	
**High blood pressure** ^**b**^									0.537
No	362 (99.7)	145 (100)	62 (98.4)	16 (100)	67 (100)	44 (100)	9 (100)	19 (100)	
Yes	1 (0.3)	0	1 (1.6)	0	0	0	0	0	
**Gestational hypertension** ^**b**^									0.866
No	358 (98.6)	143 (98.6)	62 (98.4)	16 (100)	65 (97)	44 (100)	9 (100)	19 (100)	
Yes	5 (1.4)	2 (1.4)	1 (1.6)	0	2 (3)	0	0	0	
**Heart and arterial diseases** ^**b**^									0.001
No	362 (99.7)	145 (100)	63 (100)	15 (93.8)	67 (100)	44 (100)	9 (100)	19 (100)	
Yes	1 (0.3)	0	0	1	0	0	0	0	
**Liver disease** ^**b**^									-
No	363 (100.0)	145 (100)	63 (100)	16 (100)	67 (100)	44 (100)	9 (100)	19 (100)	
Yes	0	0	0	0	0	0	0	0	
**High cholesterol** ^**b**^									0.365
No	360 (99.2)	145 (100)	61 (96.8)	16 (100)	66 (98.5)	44 (100)	9 (100)	19 (100)	
Yes	3 (0.8)	0	2 (3.2)	0	1 (1.5)	0	0	0	
**High triglycerides** ^**b**^									-
No	363 (100.0)	145 (100)	63 (100)	16 (100)	67 (100)	44 (100)	9 (100)	19 (100)	
Yes	0	0	0	0	0	0	0	0	
**Thyroid disorders** ^**b**^									0.138
No	347 (95.6)	141 (97.2)	56 (88.9)	16 (100)	64 (95.5)	42 (95.5)	9	19	
Yes	16 (4.4)	4 (2.8)	7 (11.1)	0	3 (4.5)	2 (4.5)	0	0	
**Respiratory problems** ^**b**^									0.488
No	356 (98.1)	143 (98.6)	60 (95.2)	16 (100)	66 (98.5)	44 (100)	9 (100)	18 (94.7)	
Yes	7 (1.9)	2 (1.4)	3 (4.8)	0	1 (1.5)	0	0	1 (5.3)	
**Food allergy** ^**b**^									0.125
No	356 (98.1)	144 (99.3)	62 (98.4)	15 (93.8)	63 (94)	44 (100)	9 (100)	19 (100)	
Yes	7 (1.9)	1 (0.7)	1 (1.6)	1 (6.3)	4 (6)	0	0	0	
**Cigarettes smoking before pregnancy** ^**b**^									0.856
More than 40 cigarettes	0	0	0	0	0	0	0	0	
20–40 cigarettes	4 (1.1)	2 (1.37)	1 (1.58)	0	1 (1.49)	0	0	0	
Less than 10 cigarettes	17 (4.6)	7 (4.82)	6 (9.52)	0	3 (4.47)	1 (2.27)	0	0	
No smoking	254 (69.9)	100 (68.9)	43 (68.2)	11 (68.75)	47 (70.1)	31 (70.4)	9 (100)	13 (68.4)	
choose not to respond	88 (24.24)	36 (24.8)	13 (20.6)	5 (31.25)	16 (23.8)	12 (27.2)	0	6 (31.5)	
**Cigarettes smoking during pregnancy** ^**b**^									0.398
More than 40 cigarettes	0	0	0	0	0	0	0	0	
20–40 cigarettes	1 (0.27)	0	0	0	0	1 (2.27)	0	0	
Less than 10 cigarettes	10 (2.75)	4 (2.75)	2 (3.17)	0	4 (5.97)	0	0	0	
I didn’t smoke at that time	264 (72.7)	106 (73.1)	47 (74.6)	11 (68.75)	47 (70.14)	31 (70.45)	9 (100)	13 (68.42)	
choose not to respond	88 (24.2)	35 (24.1)	14 (22.2)	5 (31.25)	16 (23.8)	12 (27.2)	0	6 (31.57)	
**e-cigarettes smoking before pregnancy** ^**b**^									0.012
More than once a day	0	0	0	0	0	0	0	0	
Once a day	3 (0.86)	0	3 (4.76)	0	0	0	0	0	
2–6 times a week	1 (0.27)	0	0	0	0	1 (2.27)	0	0	
Once a week or less	6 (1.65)	3 (2.06)	0	0	1 (1.49)	0	0	2 (10.5)	
No electronic cigarettes or other electronic nicotine products at the time	265 (73)	106 (73.10)	47 (74.6)	11 (68.75)	50 (74.6)	31 (70.4)	9 (100)	11 (57.8)	
choose not to respond	88 (24.2)	36 (24.8)	13 (20.6)	5 (31.2)	16 (23.8)	12 (27.2)	0	6 (31.5)	
**e-cigarettes smoking during pregnancy** ^**b**^									0.658
More than once a day	1 (0.27)	0	0	0	0	1 (2.27)	0	0	
Once a day	6 (1.65)	3 (2.06)	2 (3.17)	0	1 (1.49)	0	0	0	
2–6 times a week	0	0	0	0	0	0	0	0	
Once a week or less	2 (0.55)	0	0	0	1 (1.49)	1 (2.27)	0	0	
No electronic cigarettes or other electronic nicotine products at the time	267 (73.5)	107 (73.7)	48 (76.1)	11 (68.75)	49 (73.1)	30 (68.18)	9 (100)	13 (68.4)	
choose not to respond	87 (23.9)	35 (24.13)	13 (20.6)	5 (31.2)	16 (23.8)	12 (27.2)	0	6 (31.5)	
**hookah / shisha smoking before pregnancy** ^**b**^									0.71
More than once a day	10 (2.75)	3 (2.06)	3 (4.76)	0	3 (4.47)	1 (2.27)	0	0	
Once a day	27 (7.43)	9 (6.2)	3 (4.76)	1 (6.25)	7 (10.44)	2 (4.54)	3 (33.3)	2 (10.52)	
2–6 times a week	6 (1.65)	5 (3.44)	1 (1.58)	0	0	0	0	0	
Once a week or less	36 (9.91)	14 (9.65)	8 (12.69)	1 (6.25)	5 (7.46)	5 (11.36)	0	3 (15.78)	
No use of hookah / shisha at the time	198 (54.5)	79 (54.5)	35 (55.5)	9 (56.25)	37 (55.2)	24 (54.5)	6 (66.7)	8 (42.1)	
choose not to respond	86 (23.6)	35 (24.1)	13 (20.6)	5 (31.25)	15 (22.3)	12 (27.2)	0	6 (31.5)	
**hookah / shisha smoking during pregnancy** ^**b**^									0.486
More than once a day	5 (1.37)	3 (2.06)	0	0	0	2 (4.54)	0	0	
Once a day	9 (2.47)	3 (2.06)	2 (3.17)	1 (6.25)	1 (1.49)	1 (2.27)	0	1 (5.26)	
2–6 times a week	3 (0.82)	1 (0.68)	1 (1.58)	0	0	0	1 (11.1)	0	
Once a week or less	22 (6.06)	10 (6.89)	2 (3.17)	0	6 (8.95)	3 (6.8)	1 (11.1)	0	
No use of hookah / shisha at the time	238 (65.56)	93 (64.1)	45 (71.4)	10 (62.5)	45 (67.1)	26 (59)	7 (77.8)	12 (63.1)	
choose not to respond	86 (23.69)	35 (24.5)	13 (20.6)	5 (31.25)	15 (22.3)	12 (27)	0	6 (31.5)	
**Smoking pattern affected during the economic crisis** ^**b**^									0.855
No, it was not affected	197 (54.26)	77 (53.1)	40 (63.49)	7 (43.75)	38 (56.7)	23 (52.2)	6 (66.6)	6 (31.5)	
Increased	6 (1.65)	4 (2.75)	0	0	2 (2.98)	0	0	0	
Decreased	39 (10.74)	17 (11.7)	8 (12.69)	2 (12.5)	6 (8.9)	2 (4.5)	2 (22.2)	2 (10.5)	
choose not to respond	121 (33.3)	47 (32.4)	15 (23.8)	7 (43.75)	21 (31.3)	19 (43.18)	1 (11.1)	11 (57.8)	
**Supplement use** ^**b**^									0.687
Yes	161 (44.35)	61 (42)	32 (50.79)	7 (43.75)	31 (46.2)	17 (38.6)	4 (44.4)	9 (47.3)	
No	7 (1.92)	1 (0.68)	1 (1.58)	1 (6.25)	2 (2.98)	1 (2.27)	0	1 (5.26)	
choose not to respond	195 (53.7)	83 (57.2)	30 (47.6)	8 (50)	34 (50.7)	26 (59)	5 (55.5)	9 (47.3)	
**PA before pregnancy** ^**b**^									0.380
Not Active before pregnancy	61 (16.8)	25 (17.2)	15 (23.8)	3 (18.75)	7 (10.44)	6 (13.6)	2 (22.2)	3 (15.7)	
Active before pregnancy	103 (28.3)	35 (24.1)	16 (25.3)	5 (31.2)	26 (38.8)	12 (27.2)	2 (22.2)	7 (36.8)	
choose not to respond	199 (54.8)	85 (58.6)	32 (50.7)	8 (50)	34 (50.7)	26 (59)	5 (55.5)	9 (47.3)	
**PA during pregnancy** ^**b**^									0.322
Not Active during pregnancy	74 (20.38)	29 (20)	17 (26.9)	4 (25)	10 (14.9)	6 (13.6)	3 (33.3)	5 (26.3)	
Active during pregnancy	90 (24.79)	31 (21.3)	14 (22.2)	4 (25)	23 (34.3)	12 (27.2)	1 (11.1)	5 (26.3)	
choose not to respond	199 (54.8)	85 (58.6)	32 (50.79)	8 (50)	34 (50.7)	26 (59)	5 (55.5)	9 (47.3)	
**Change in PA pattern during the economic crisis** ^**b**^									0.177
Increased	11 (3.03)	3 (2)	0	1 (6.25)	1 (1.4)	4 (9)	0	2 (10.5)	
Decreased	123 (33.8)	46 (31.7)	23 (36.5)	5 (31.25)	28 (41.17)	12 (27.2)	3 (33.3)	6 (31.5)	
No Change	30 (8.2)	11 (7.5)	8 (12.68)	2 (12.5)	4 (5.9)	2 (4.5)	1 (11.1)	2 (10.5)	
choose not to respond	199 (54.8)	85 (58.6)	32 (50.7)	8 (50)	34 (50.7)	26 (59)	5 (55.5)	9 (47.3)	

^a^ Statistical test used: One-way ANOVA

^b^ Statistical test used: Chi-square. P<0.05 was considered significant.

### Dietary patterns of pregnant women

Table 2 compares serving intakes and compliance with USDA dietary recommendations over the two time periods—prior to and throughout the socioeconomic crisis. While following the USDA guidelines did not change much prior to or in the socioeconomic crisis, the mean intake of vegetables changed slightly (p = 0.025). The intake of all food groups (Cereals, Vegetables, Fruits, Dairy and Proteins Group) was below the USDA recommendations among the majority of our sample. This was thus reflected in the total score of adherence to USDA recommendations, which was low among the vast majority.

**Table 2:** Adherence to USDA dietary recommended intakes, per servings, among the total sample before and during the socioeconomic crisis (N = 124).

	Before crisis	During crisis	p-value
Cereals (mean±SD)^a^	**1.94±2.85**	**2.09±3.27**	**0.112**
Cerealsb			**1.00**
<6 servings/day	**117 (94.4)**	**117 (95.1)**	
≥6 servings/day	**7 (5.6)**	**6 (4.9)**	
Vegetables (mean±SD) ^a^	**1.59±1.55**	**1.72±1.87**	**0.025**
Vegetablesb			**1.00**
<2.5 servings/day	**98 (79)**	**97 (78.2)**	
≥2.5 servings/day	**21 (16.9)**	**20 (16.12)**	
Choose not to respond	**5 (4)**	**7 (5.6)**	
Fruits (mean±SD) ^a^	**1.68±1.48**	**1.82±1.75**	**0.091**
Fruitsb			**0.064**
<2 servings/day	**76 (61.2)**	**66 (53.2)**	
≥2 servings/day	**46 (37)**	**54 (43.5)**	
Choose not to respond	**2 (1.6)**	**4 (3.22)**	
Dairy (mean±SD) ^a^	**1.25±1.19**	**1.25±1.12**	**0.790**
Dairyb			**1.00**
<3 servings/day	**111 (89.5)**	**109 (87.9)**	
≥3 servings/day	**7 (5.6)**	**7 (5.6)**	
Choose not to respond	**6 (4.83)**	**8 (6.45)**	
Protein group (mean±SD) ^a^	**2.28±2.58**	**2.25±2.28**	**0.874**
Proteinb			**1.00**
<5.5 servings/day	**117 (94.3)**	**115 (92.7)**	
≥5.5 servings/day	**4 (3.22)**	**4 (3.22)**	
Choose not to respond	**3 (2.41)**	**5 (4)**	
Adherence to USDA recommendations (n = 355) b			**1.00**
0–2 (Low Adherence)	**119 (33.5)**	**118 (33.2)**	
3–5 (High Adherence)	**8 (2.25)**	**8 (2.25)**	
Choose not to respond	**228 (64.2)**	**229 (64.5)**	

^a^ non-parametric Related-samples Wilcoxon Signed Rank Test

^b^ non-parametric Related-samples McNemar Test; p<0.05 is significant.

S1 and S2 Tables in supplementary material represent the adherence to the USDA recommended servings intake across governorates. Findings were comparable across governorates and showed similar results: the adherence to USDA recommendations was low among the vast majority across governorates, with the intakes of food groups being below the USDA recommendations. In contrast, fruits in particular showed a more even distribution as per the adherence to recommendations in South governorate. In line with the findings among the total sample, dietary intakes before the crisis did not remarkably change as compared to those during the crisis.

Table 3 shows the dietary patterns of pregnant women as assessed by the adherence to USDA dietary recommended intakes, according to dietary habits changes throughout the socioeconomic crisis. The mean adherence level was significantly higher among participants who reported following a popular diet that is popular among social interactors. The adherence level differed across other dietary patterns, nevertheless these differences were not statistically significant. The dietary habits variations among pregnant women analyzed that were non statistically significantly associated with a higher USDA dietary recommended intake adherence average (p>0.05; Table 3) include the following: intake of more than 3 meals/day (p = 0.162), overall impact on food pattern (p = 0.656) and food intake times/day (p = 529) with higher amounts consumed (p = 0.727). Indeed as Table 3 shows pregnant women with: worsened food quality (p = 0.488), a predominance of cheaper foods (p = 0.966) intake, a non-reduction of meals number even though there is not enough food (p = 0.884) had a superior adherence score to the USDA dietary recommended intake. Moreover, it is also interesting to note that the USDA dietary adherence average was non statistically significantly higher among pregnant women who preferred easy to prepare food (p = 0.851), those eating more foods requiring less ingredients (p = 092), those who increased their consumption of healthier foods (p = 0.565) with a higher nutrient-density (p = 0.338).

**Table 3:** The dietary patterns as assessed by the adherence to USDA dietary recommended intakes of pregnant women according to dietary habits changes throughout the socioeconomic crisis.

	Mean	SD	SE	p-value
**Main meals (breakfast—lunch—dinner) eaten per day**^**a**^ **(N = 168)**				0.162
One meal per day	0.00	0.00	0.00	
Two meals a day	0.59	0.57	0.11	
Three meals a day	0.84	1.18	0.12	
**Food pattern affected**^**b**^ **(N = 168)**				0.656
Yes	0.86	1.42	0.24	
No	0.72	0.90	0.09	
**Eating times throughout the day affected** ^**b**^ ^**(**^**N = 168)**				0.529
Yes	1.03	1.51	0.26	
No	0.66	0.84	0.09	
**Amount of food eaten per day affected** ^**a**^ **(N = 168)**				0.727
The amount of food eaten has increased	0.95	1.35	0.22	
having less food	0.75	1.18	0.30	
Not affected	0.67	0.86	0.10	
**Worsened quality of food** ^**b**^ **(N = 363)**				0.488
No	0.75	1.07	0.10	
Yes	0.91	1.14	0.34	
**Going to cheaper foods** ^**b**^ **(N = 363)**				0.906
No	0.75	1.04	0.10	
Yes	0.81	1.18	0.23	
**Reducing the number of meals because there is not enough food** ^**b**^ **(N = 363)**				0.884
No	0.77	1.08	0.10	
Yes	0.50	0.71	0.50	
**Go to easy-to-prepare foods such as sandwiches, French fries, etc** ^**b**^ ^**(**^**N = 363)**				0.851
No	0.75	1.02	0.09	
Yes	1.00	1.83	0.69	
**Go to foods that do not require much food when preparing them** ^**b**^ **(N = 363)**				0.092
No	0.74	1.11	0.10	
Yes	0.92	0.64	0.18	
**Reducing the intake of vegetables, fruits, meat and dairy products due to lack of financial means)** ^**b**^ **(N = 363)**				0.565
No	0.77	1.06	0.10	
Yes	0.70	1.25	0.40	
**Increased your consumption of healthy foods and foods with high nutritional value**^**b**^ **(N = 168)**				0.338
Yes	0.86	1.19	0.13	
No	0.58	0.78	0.12	
**Statement that best describes the diet** ^**a**^ **(N = 168)**				0.020
Eating a balanced diet rich in fruits and vegetables, whole grains, and legumes	0.68	1.00	0.10	
Eating an unhealthy diet rich in fats, sugars, sweets and processed meats	0.50	0.53	0.19	
Following a popular diet that is popular among social interactors	3.00	1.83	0.91	

SD: Standard Deviation; SE: Standard Error

^a^ Non-parametric Independent-samples Kruskal-Wallis Test

^b^ Non-parametric Independent-samples Mann-Whitney U Test; p<0.05 is significant.

## Discussion

The findings of this study unveiled low adherence to USDA recommendations both before and during the socioeconomic crisis that impacted Lebanon. These results are alarming as they raise concerns about the health of Lebanese pregnant women; such low adherence translates into potential deficiencies in key nutrients, thereby compromising nutritional status. This, in turn, impacts fetal health and development, and increases the likelihood of gestational complications for mothers.

This low adherence could be caused by the nutrition transition trend occurring in Lebanon and neighboring countries. As a matter of fact, recent findings showed that only a small proportion of the Lebanese population has a high compliance to the Mediterranean Diet, with a dominance of a Westernized dietary pattern, due to the ongoing nutrition transition in Lebanon [13, 14]. Another reason could be the specific dietary habits of pregnant women. In fact, a systematic review showed that pregnant women in most studies reported a low adherence to USDA dietary recommendations for vegetables and grains/cereals; however, they were adherent to dairy and fruit intake [15]. Factors that were shown to be associated with low adherence to USDA guidelines included a lower education level, a lower age, and smoking [15]. It is crucial for pregnant women to eat “right” during pregnancy, while also avoiding unbalanced restrictive dieting. Maternal diet closely affects the child’s health, and has been associated with a higher risk of stunting [16–18]. In this respect, a specific driver of malnutrition and stunting is a low socioeconomic status [19]. Healthy eating is not only essential for an optimal fetal growth, but also for a better health for the mother, hence protecting her from severe complications such as pre-eclampsia [5, 20].

The adherence level did not differ before and after the socioeconomic crisis. Compared with studies conducted during Covid-19 pandemic that had a substantial socioeconomic impact on all countries, findings are in line with a study on Spanish pregnant women that reported there was no change in the participants’ dietary habits [21]. Nevertheless, while around 17% of women claimed that their eating habits became worse, 42% showed enhancements and 41% recorded no shifting, according to another US study [22]. While the adherence level to USDA guidelines did not significantly differ between pre- and post-crisis periods, consumption of vegetables was increased. This is in line with studies reporting healthier dietary habits among pregnant women as compared to before pregnancy, including a rise in the fruits and vegetables intakes [23]. Another study among pregnant individuals in Canada showed that women reported the intake of greater quantities of fresh vegetables, fruits, and snacks, while consuming less junk food and takeaway or home delivery. In Lebanon, although data is scarce on pregnant women dietary changes during the socioeconomic crisis, a study conducted on Lebanese pregnant women determined 3 dietary patterns, with one of them being the “Mixed Dietary Pattern”, characterized by an intake of nutritious dietary choices (fruits, vegetables, seeds) paired with Western diet (Lebanese street foods)” [24]. In this study, the other 2 dietary patterns were however the “Westernized dietary pattern”, and the “Neo-Mediterranean” dietary pattern characterized by “protein-based choices” [24].

Regarding physical activity, while the proportion of active women slightly decreased during the socioeconomic crisis, the majority was still active. A recent study among adults in Lebanon showed a decline in workouts after the pandemic [25]. In the US study, 22% reported being inactive, 2% became active, and 76% showed no change [22]. When there are no counter indications, physical activity and exercise during pregnancy are highly beneficial, and have been associated with a lower incidence of gestational diabetes, preterm birth, and of lower birth weight [26].

Limitations of this study include the online sampling method, leading to unbalanced stratification across governorates, therefore preventing more detailed analysis between these areas. In addition, there is a recall bias particularly towards food consumption patterns before the socioeconomic crisis. Nevertheless, this data collection method allowed a fast and easy way to reach the sample. Moreover, this study provided important insights on the eating habits of expectant women in Lebanon after the socioeconomic crisis and this highlights the significance of carrying out specific actions to enhance the health of such a critical group.

## Conclusion

Findings of this study showed a low adherence to the USDA recommended consumptions of Lebanese pregnant women throughout the socioeconomic crisis, without notable differences in comparison to the pre-crisis period. Higher attention should be given to the dietary habits and health of this critical population, through effective interventions that increase awareness and achieve measurable improvements.

## Supporting information

S1 Table Adherence to USDA dietary recommendations before the socioeconomic crises. (DOCX)

S2 Table Adherence to USDA dietary recommendations during the socioeconomic crises. (DOCX)
