# Supplementary material for: Assessment of dietary-lifestyle patterns and adherence to the USDA recommendations in Lebanese pregnant women amid the economic crisis: Findings from a national representative cross-sectional study
Source: PLoS One. 2024 Apr 11;19(4):e0301170. doi: 10.1371/journal.pone.0301170 (PMC11008882; doi:10.1371/journal.pone.0301170)
Supplement: S1 Table — (DOCX) [file pone.0301170.s001.docx]

**Table S1**. **Adherence to USDA dietary recommendations before the socioeconomic crises.**

|  | **Governorates** | | | | | | |  |
| --- | --- | --- | --- | --- | --- | --- | --- | --- |
|  | **Beirut** | **Mount Lebanon** | **North Lebanon** | **South Lebanon** | **Nabatieh** | **Beqaa** | **Baalbek-Hermel** | **p-value** |
| **Cereals** |  |  |  |  |  |  |  | 0.044 |
| <6 servings/day | 100 (91.7) | 92 (96.8) | 14 (100.0) | 72 (100.0) | 38 (100.0) | 7 (100.0) | 16 (100.0) |  |
| ≥6 servings/day | 9 (8.3) | 3 (3.2) | 0 (0.0) | 0 (0.0) | 0 (0.0) | 0 (0.0) | 0 (0.0) |  |
| **Vegetables** |  | | | | | | | 0.809 |
| <2.5 servings/day | 94 (89.5) | 80 (84.2) | 12 (92.3) | 59 (84.3) | 28 (80.0) | 6 (85.7) | 14 (87.5) |  |
| ≥2.5 servings/day | 11 (10.5) | 15 (15.8) | 1 (7.7) | 11 (15.7) | 7 (20.0) | 1 (14.3) | 2 (12.5) |  |
| **Fruits** |  |  |  |  |  |  |  | 0.824 |
| <2 servings/day | 71 (65.1) | 66 (71.0) | 9 (69.2) | 40 (58.8) | 23 (63.9) | 5 (71.4) | 11 (68.8) |  |
| ≥2 servings/day | 38 (34.9) | 27 (29.0) | 4 (30.8) | 28 (41.2) | 13 (36.1) | 2 (28.6) | 5 (31.3) |  |
| **Dairy** |  |  |  |  |  |  |  | 0.796 |
| <3 servings/day | 99 (90.8) | 87 (93.5) | 12 (100.0) | 65 (95.6) | 33 (91.7) | 6 (85.7) | 15 (93.8) |  |
| ≥3 servings/day | 10 (9.2) | 6 (6.5) | 0 (0.0) | 3 (4.4) | 3 (8.3) | 1 (14.3) | 1 (6.3) |  |
| **Protein** |  |  |  |  |  |  |  | 0.307 |
| <5.5 servings/day | 98 (89.1) | 88 (92.6) | 13 (100.0) | 68 (97.1) | 36 (97.3) | 6 (85.7) | 15 (93.8) |  |
| ≥5.5 servings/day | 12 (10.9) | 7 (7.4) | 0 (0.0) | 2 (2.9) | 1 (2.7) | 1 (14.3) | 1 (6.3) |  |
| **Adherence score** |  |  |  |  |  |  |  | 0.349 |
| 0-2 (Low Adherence) | 105 (92.9) | 89 (91.8) | 14 (100.0) | 71 (98.6) | 38 (97.4) | 6 (85.7) | 15 (93.8) |  |
| 3-5 (High Adherence) | 8 (7.1) | 8 (8.2) | 0 (0.0) | 1 (1.4) | 1 (2.6) | 1 (14.3) | 1 (6.3) |  |

Statistical test: Chi-Square; p<0.05 is considered significant
