# Supplementary material for: Assessment of dietary-lifestyle patterns and adherence to the USDA recommendations in Lebanese pregnant women amid the economic crisis: Findings from a national representative cross-sectional study
Source: PLoS One. 2024 Apr 11;19(4):e0301170. doi: 10.1371/journal.pone.0301170 (PMC11008882; doi:10.1371/journal.pone.0301170)
Supplement: S2 Table — (DOCX) [file pone.0301170.s002.docx]

**Table S2**. **Adherence to USDA dietary recommendations during the socioeconomic crises.**

|  | Governorates | | | | | | | |
| --- | --- | --- | --- | --- | --- | --- | --- | --- |
|  | **Beirut** | **Mount Lebanon** | **North Lebanon** | **South Lebanon** | **Nabatieh** | **Beqaa** | **Baalbek-Hermel** | **p-value** |
| **Cereals** |  |  |  |  |  |  |  | 0.062 |
| <6 servings/day | 98 (91.6) | 93 (97.9) | 14 (100.0) | 70 (98.6) | 38 (100.0) | 7 (100.0) | 16 (100.0) |  |
| ≥6 servings/day | 9 (8.4) | 2 (2.1) | 0 (0.0) | 1 (1.4) | 0 (0.0) | 0 (0.0) | 0 (0.0) |  |
| **Vegetables** |  |  |  |  |  |  |  | 0.972 |
| <2.5 servings/day | 89 (84.0) | 77 (82.8) | 11 (84.6) | 59 (84.3) | 29 (82.9) | 6 (85.7) | 15 (93.8) |  |
| ≥2.5 servings/day | 17 (16.0) | 16 (17.2) | 2 (15.4) | 11 (15.7) | 6 (17.1) | 1 (14.3) | 1 (6.3) |  |
| **Fruits** |  |  |  |  |  |  |  | 0.693 |
| <2 servings/day | 65 (60.2) | 62 (67.4) | 9 (69.2) | 40 (58.8) | 20 (55.6) | 5 (71.4) | 12 (75.0) |  |
| ≥2 servings/day | 43 (39.8) | 30 (32.6) | 4 (30.8) | 28 (41.2) | 16 (44.4) | 2 (28.6) | 4 (25.0) |  |
| **Dairy** |  |  |  |  |  |  |  | 0.217 |
| <3 servings/day | 92 (85.2) | 86 (93.5) | 12 (100.0) | 62 (91.2) | 35 (97.2) | 6 (85.7) | 15 (93.8) |  |
| ≥3 servings/day | 16 (14.8) | 6 (6.5) | 0 (0.0) | 6 (8.8) | 1 (2.8) | 1 (14.3) | 1 (6.3) |  |
| **Protein** |  |  |  |  |  |  |  | 0.533 |
| <5.5 servings/day | 98 (90.7) | 87 (93.5) | 13 (100.0) | 66 (94.3) | 36 (97.3) | 6 (85.7) | 16 (100.0) |  |
| ≥5.5 servings/day | 10 (9.3) | 6 (6.5) | 0 (0.0) | 4 (5.7) | 1 (2.7) | 1 (14.3) | 0 (0.0) |  |
| **Adherence** |  |  |  |  |  |  |  | 0.412 |
| 0-2 (Low Adherence) | 99 (89.2) | 90 (92.8) | 14 (100.0) | 68 (95.8) | 38 (97.4) | 6 (85.7) | 15 (93.8) |  |
| 3-5 (High Adherence) | 12 (10.8) | 7 (7.2) | 0 (0.0) | 3 (4.2) | 1 (2.6) | 1 (14.3) | 1 (6.3) |  |

Statistical test: Chi-Square; p<0.05 is considered significant
